# Targeting bacterial degradation machinery as an antibacterial strategy

**DOI:** 10.1042/BCJ20230191

**Published:** 2023-11-02

**Authors:** Radoslav Petkov, Amy H. Camp, Rivka L. Isaacson, James H. Torpey

**Affiliations:** 1 Department of Chemistry, King's College London, Britannia House, 7 Trinity Street, London SE1 1DB, U.K.; 2 Department of Biological Sciences, Mount Holyoke College, 50 College Street, South Hadley, Massachusetts 01075, U.S.A.

**Keywords:** AAA proteins, antibiotic resistance, degradation, PROTACs

## Abstract

The exploitation of a cell's natural degradation machinery for therapeutic purposes is an exciting research area in its infancy with respect to bacteria. Here, we review current strategies targeting the ClpCP system, which is a proteolytic degradation complex essential in the biology of many bacterial species of scientific interest. Strategies include using natural product antibiotics or acyldepsipeptides to initiate the up- or down-regulation of ClpCP activity. We also examine exciting recent forays into BacPROTACs to trigger the degradation of specific proteins of interest through the hijacking of the ClpCP machinery. These strategies represent an important emerging avenue for combatting antimicrobial resistance.

## Introduction

The antibiotic resistance crisis is one of the top priorities for the scientific community in the 21st century and poses a major threat to human health and food security. There are numerous types of antibiotics including, but not limited to: beta lactams, which bind to the transpeptidases needed for cell wall biosynthesis; glycopeptides, which inhibit peptidoglycan production by targeting lipid II; aminoglycosides, which disrupt protein synthesis through binding to the ribosomal 30S subunit; and quinolones, which target DNA synthesis and replication through the inactivation of DNA gyrase and topoisomerase IV [1]. Further detail into the mechanisms of action of the different classes of antibiotics is beyond the scope of this review, however there exist other fantastic reviews into this topic [2,3]. The beta lactam antibiotics were the first antibiotics to be developed and resistance to this class of antibiotics is now widespread. This resistance is mediated through the use of efflux pumps to remove the antibiotics from the cell, by modifying the target transpeptidases, and through the use of beta lactamases that will degrade them [1,4,5]. Similarly, elaborate resistance mechanisms have also emerged for the newer, more recent classes of antibiotics. As a consequence of this, in the last decade scientists have had to diversify their antimicrobial strategies.

**Figure 1.**
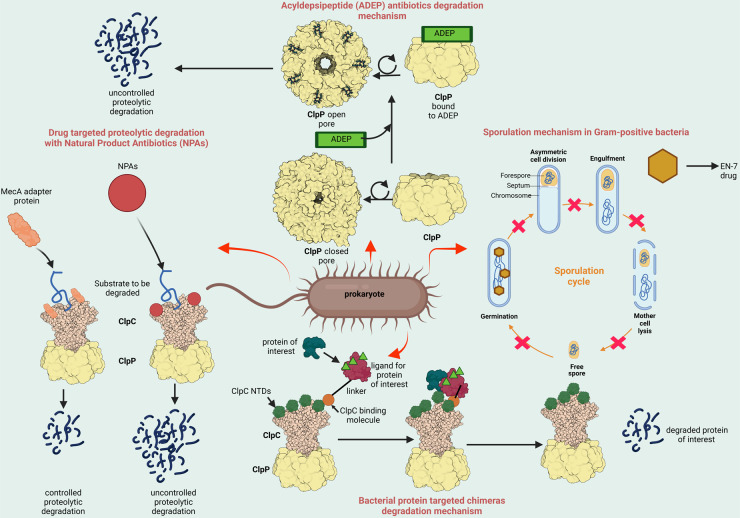
Summary diagram of the compounds designed to target the targeted protein degradation machinery in bacteria. Figure is adapted from the following references and generated in BioRender [34,46,49].

**Figure 2.**
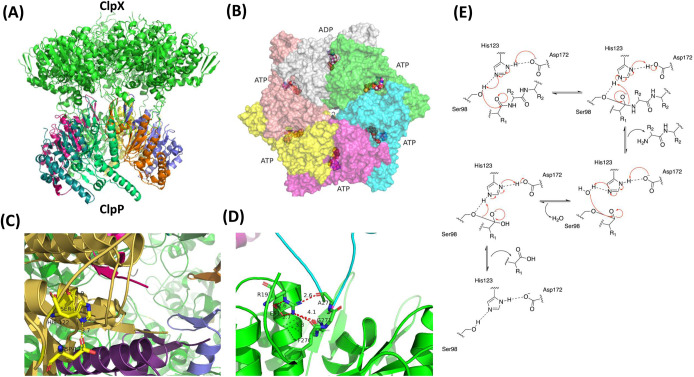
Structure of the ATPase ClpP proteolytic degradation system in bacteria and its serine protease degradation mechanism [7,8]. (**A**) The hexameric ClpX ATPase bound to the tetradecameric ClpP peptidase in *E.coli* bacteria (PDB: 6PO3). Each of the seven sub-units in ClpP is represented in a different colour. (**B**) Top view of hexameric ClpX with the six different protomers responsible for substrate binding, unfolding and translocation shown in different colours. (**C**) Catalytic triad in ClpP comprising Ser98, His123 and Asp172; (**D**) IGF/L binding region showing the key amino acids responsible for binding of ClpX with ClpP. For diagrams (**C**) and (**D**) red dashed lines show hydrogen bonding. Green and yellow colour was used to represent carbon and conventional colour scheme was used to represent nitrogen and oxygen. (**E**) Chemical mechanism of protein degradation by catalytic triad in ClpP.

One such strategy is by harnessing the bacterium's own internal protein degradation machinery to kill them (Figure 1). This idea was first conceived in 1997 following the structural determination of the caseinolytic protease P (ClpP) from *Escherichia coli* (*Ec*) [6]. Bacteria use this proteolytic complex and many other peptidases to degrade proteins of interest. ClpP operates in conjunction with one of several AAA+ ATPase proteins that usually take the form of a hexameric ring and serve the role of unfolding protein substrates prior to degradation. AAA+ proteins are a large superfamily of proteins found across life that are employed in a wide range of roles, including in cell division, modulating stress response, virulence, antibiotic resistance and many others [7]. The Clp system contains a multitude of AAA+ ATPase proteins, which form an active complex with a protease component (e.g. ClpP) (Figure 2). ClpP is composed of two stacks of heptameric rings, which combine to form a tetradecameric barrel-like structure [8]. The β catalytic site is composed of 14 serine proteases (catalytic triads of ser98-his123-asp172) sandwiched between two α helices [8–9]. These peptidases remain inactive and can only degrade very small peptides diffusing passively into the structure and require chaperone proteins for their activation which connect to the ClpP through Ile-Gly-Phe/Leu (IGF/L) loops [10] (Figure 2). ClpP has several regulatory ATPase partners, which unfold proteins and prepare them for degradation, such as ClpX and ClpA in Gram-negative species (e.g. *E. coli*), ClpC and ClpE in Gram-positive species (e.g. *Bacillus subtilis* (*Bs*)), and ClpC1 in *Mycobacterium tuberculosis* (*Mtb*) [11]. Each monomer of these hexameric proteins contains a Walker A and Walker B motif (required for ATP binding and hydrolysis, respectively) to provide the mechanical energy for protein binding, unfolding and translocation into the peptidase where the protein is degraded [12]. In bacteria, multiple ring proteases including: ClpP, Lon, ClpYQ (HsIUV), FtsH, associate with various AAA+ ATPases to degrade proteins however ClpP has been most widely studied [7]. Many species of bacteria, such as *B. subtilis*, require adaptor proteins that regulate the activity of the ATPase-protease complex. These adaptor proteins enable the ATPase and ring protease to form an active complex and facilitate the processing of specific substrates [13,14]. For example *B. subtilis* utilises MecA to trigger ClpCP-mediated degradation of ComK in competence development, while ClpB in *E.coli* cooperates with DnaK, DnaJ and GrpE to promote protein disaggregation and refolding [15]. In summary, the Clp system is highly regulated to ensure only the correct proteins are targeted, as inappropriate protein degradation may lead to bacterial cell death. This review will examine drugs that have been developed to modulate the proteolytic degradation machinery in bacteria to combat the growing threat of antimicrobial resistance (AMR).

## Bacterial ATPases target substrates for proteolytic degradation

In contrast with the eukaryotic proteasome, bacteria have an array of different components, which offer lots of scope for drug development. One such example is ClpYQ. This is composed of the AAA+ ATPase hexamer HsIU_6_ and the HsIUV_12_ protease and is activated during heat shock in *E. coli*. At low temperatures the HsIU_6_ hexamer forms a trimeric plug, blocking protein entry and at high temperature the plug becomes deactivated allowing for protein entry and subsequent degradation [16]. The heat shock-activated mechanism of this complex in *E. coli* was believed to apply in other bacterial species, however it was found that in Gram-positive species, including *B. subtilis* and *B. cereus*, the protein was not heat shock responsive [17]. In addition, differences in the active sites between the Gram-positive and Gram-negative bacteria mean that the antibiotic design must be tailored to the different species [17]. The mechanism of action of these proteins is still not fully understood, hence antibiotic development to target these proteins is in its infancy, however, future antibiotics could be made to change the hydrophobic packing on the surface of HsIU_6_ hexamer, which is believed to be necessary for protein interaction [16].

Lon proteases are a diverse class of proteins found in prokaryotic and eukaryotic species. The ATPase domain consists of six protomers, where four arranged in a ‘spiral staircase' arrangement bind to an incoming substrate while two remain unbound making up the so-called ‘seam' [18]. Following binding, the substrate is then unfolded and translocated — via an ATP-driven processive rotary mechanism — into the protease domain of Lon where it is degraded. This mechanism is also shared by other AAA+ ATPases [19]. There remains uncertainty regarding the identity of the substrates involved in Lon-mediated degradation. A recent proteomics study suggested that Lon may degrade key proteins in the cell cycle including: Ccrm, SciP, DnaA, FliK and StaR [20]. However, the current lack of understanding regarding its mechanism of action and its precise targets make Lon an unsuitable target for antibiotic development at present. In addition, it has been found that many small molecule inhibitors designed for Lon also target the 20S proteosome, which means potential antibiotics from these inhibitors could result in off-target effects [21]. Recent studies have been able to synthesise a small molecule peptide inhibitor pyrazinamide-l-homoArg-neopentylGly-Leu-B(OH)_2_ that has been found to selectively target Lon because of the homoarginine present [21]. Hence, boronic acid inhibitors with the homoarginine could be investigated for future antibiotic development.

ClpP, on the other hand, has been the subject of many studies over the last 20 years. Structural characterisation studies of ClpC and ClpP in different bacterial species was initially difficult to perform, because of the large size of the proteins, as well as the presence of flexible loops in conjunction with conformational heterogeneity and low water solubility. These factors make these systems challenging to probe via X-ray crystallography and require the removal of the loops, rendering the systems inactive [14]. Cryo-EM post ‘resolution revolution' has provided much new insight into this family of proteins.

Cryo-EM studies of these ATPases have now identified structural, conformational and substrate binding features. In *Staphylococcus aureus* (*Sa*) ClpC is found to exist in an inactive decamer state when no adaptor protein is present and active hexamer state when adaptor protein MecA is present. Cryo-EM studies, have confirmed that the inactive state is mediated by intramolecular assembly of middle domain (MD) regions preventing substrate binding and protecting the cell from unnecessary protein degradation [22]. Mutation of key residues such as Phe436 in ClpC in *S.aureus*, ClpL and ClpE in other bacterial species shows high levels of toxicity as uncontrolled protein degradation takes place, since the MD region can no longer associate [22]. In addition, the N-terminal domain (NTD) region of the proteins have been well characterised, using X-ray crystallography and NMR solution studies, which has elucidated the binding region of natural product antibiotics (NPAs) with the NTD [23]. Recently, several structures of the ClpXP complex were determined by cryo-EM. These employed several strategies, including the utilisation of slowly hydrolysable ATPγS and, in one instance, a single chain variant of ClpX lacking catalytic activity [10,24,25]. These degradative systems may be exploited as a viable antibiotic target.

## Innovative technologies with PROTACs, for targeted proteolytic degradation

**Figure 3.**
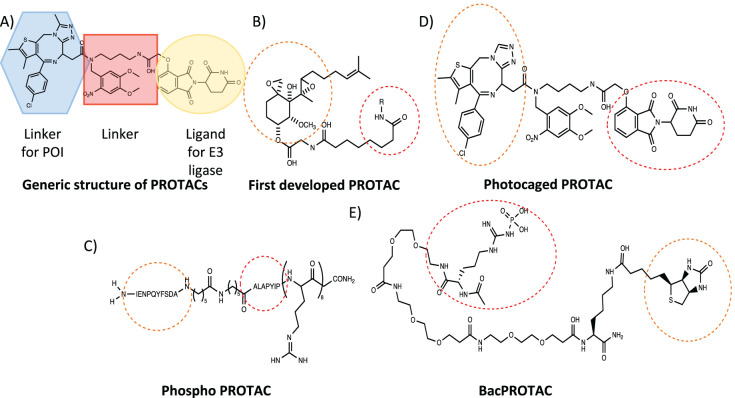
Variety of different PROTACs developed over the last decade. (**A**) This shows the basic elements of any PROTAC: the ligand for the given protein of interest (blue), the linker (red), and the ligand region targeted to the E3 ubiquitin ligase (yellow) [29]. (**B**) The first developed PROTAC that targeted the methionine aminopeptidase-2 (MetAP-2) to the Skp1-Cullin-F box (SCF) complex containing Hrt1 for ubiquitination and degradation [29]. (**C**) A photocaged PROTAC that triggers light-induced degradation of Brd4 protein via linkage to an E3 ubiquitin ligase [33]. (**D**) A phosphoPROTAC that couples a ligand-binding domain for a RTK with a peptide ligand for the von Hippel Lindau E3 ubiquitin ligase [32]. (**E**) A BacPROTAC designed to target monomeric streptavidin (mSA) to the bacterial ClpCP degradation machinery [34]. Orange dotted circles represent the ligand designed to recruit the specific protein of interest (POI). Red dotted circles represent the ligand or peptide sequence responsible for recruiting the E3 ligase or ClpCP in the case of BacPROTACs. All diagrams were drawn using Chemdraw software.

A recent strategy has involved recruiting the proteolytic degradation machinery to destroy specific targets. This has led to the development of proteolysis-targeting chimeras (PROTACS) [26]. PROTACs were created for specific proteolytic degradation in eukaryotic cells, by manipulating the ubiquitin proteasomal pathway. This begins with a reaction cascade orchestrated by three enzymes: the E1 ubiquitin activating enzyme, E2 which transfers ubiquitin from E1 to E3, and E3 which is responsible for attaching a ubiquitin to the lysine of the protein to be degraded [27,28]. This process is repeated to form a polyubiquitin chain, acting as a tag for delivery to the 26S proteasome for protein degradation. PROTACs usually contain a ligand specific to the protein of interest (POI), a ligand specific to an E3 ligase and a linker to connect the two sections together (Figure 3A).

The first PROTAC to be designed by *Sakamoto et al*. contained an ovalicin ligand, which is an inhibitor targeting the POI MetAP-2 and a ubiquitinated protein Skp1-Cullin-F E3 ligase [29] (Figure 3B). The PROTAC contained a peptide chain recognised by the E3 ligase, which facilitated protein ubiquitination, however it was concluded that cell permeability was poor with the first generation PROTACs [29]. Although there are peptide-based antibiotics with improved physiochemical properties (such as phosphoPROTACs), the focus in the field has shifted in the last decade to small molecule ligands, because of greater stability and improved physiochemical properties *in vivo* [26].

PROTACs have been widely exploited due to their many advantages relative to small molecule inhibitors. These advantages include better drug target specificity, the need for only substoichiometric quantities of PROTAC, and their ability to target ‘undruggable' proteins [30]. Recent efforts have been using PROTACs to target the phosphotyrosine signalling pathway. When receptor tyrosine kinases (RTKs) are activated, they become phosphorylated and lead to the phosphorylation of downstream protein targets, which contain a phosphotyrosine binding domain (PTB) and a Src homology 2 (SH2) domain [31]. This has triggered the development of phosphoPROTACs. These possess a kinase substrate in an inactive unphosphorylated state, which upon activation and phosphorylation by the RTK can bind to a kinase with PTB or SH2 domains and inhibit the signalling pathway [32] (Figure 3C). The designed phosphoPROTACs have been shown to inhibit the protein FRS2α [32]. This can prevent further cascading reactions, such as the activation of MARP protein, which has been successfully used to prevent tumour growth and demonstrates that these compounds show potential for cancer treatment [32]. Expanding further on the PROTAC principle, new developments include: photocaged PROTACs, phosphoPROTACs, O'PROTACs and BacPROTACs [32–35] (Figure 3).

One of the problems of PROTACs development has been the E3 ligase substrates. Even though there are 600 known E3 ligase substrates, very few have been explored due to the lack of known ligands [30]. In addition, very few studies have investigated the metabolism of these molecules after they have successfully mediated degradation of their target. A recent paper by *Goracci et al*. concluded that shorter linker PROTACs, and those with cyclic linkers, have a longer life-time before degradation [36]. Linker attachment points are also likely to be important in metabolising these compounds.

There exist numerous excellent reviews on this topic [37–39].

## Novel PROTACs targeting bacteria

**Figure 4.**
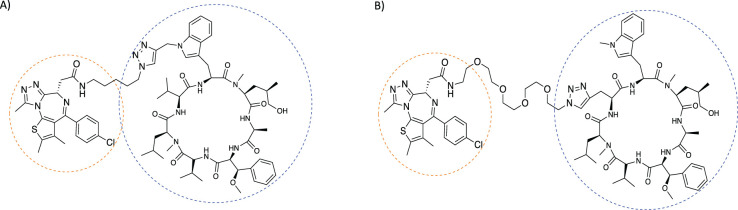
BacPROTACs constituted of Cyclomarin A (blue) and JQ1 ligand (orange) [34]. (**A**) BacPROTAC with linker attachment point on valine. (**B**) BacPROTAC with linker attachment point on tryptophan. Diagrams were drawn using ChemDraw software.

The first PROTACs designed for use in bacteria were aimed at the proteolytic ClpCP system in *B.subtilis*, and have been dubbed BacPROTACs [34]. Since bacteria do not utilise the ubiquitin proteasomal pathway the phosphoarginine group incorporated acts as a natural degron recognised by the ClpCP system [40]. This degron binds to the N-terminal domain of ClpC (ClpC_NTD_) which in-turn is recognised by the ClpP protease (Figure 3E). Cryo-EM revealed that binding of BacPROTACs to Sa-ClpC_NTD_ induced the inactive decameric state of ClpC1 to form a proteolytically active ‘tetramer of hexamers' active state [34]. However, because the phosphoarginine group suffers from poor pharmacokinetic properties, the study instead used BacPROTACs containing the antibiotic peptide Cyclomarin A [34]. In addition, to expand on the POIs, an alternative ligand JQ1 was used which binds to bromodomain-containing proteins [41]. By linking JQ1 to a cyclomarin A-like peptide *M. smegmatis* growth was successfully inhibited, through the targeted degradation of BRDT by ClpCP. Tandem mass tag spectrometric analysis suggested that this degradation was highly specific [34]. Whilst linker length did not play an important role in the activity of the BacPROTAC, *in vivo* studies in mycobacteria showed that even slight changes in the linker attachment points could cause a 15-fold increase in degradation of the model substrate, ThrC. This suggests that experimental studies should be careful when comparing *in vitro* and *in vivo* data [34] (Figure 4).

Whilst this study was successful, it was based on the well-characterised JQ1 ligand and further work is required.

## Natural product antibiotics targeting ATPases in bacteria

**Figure 5.**
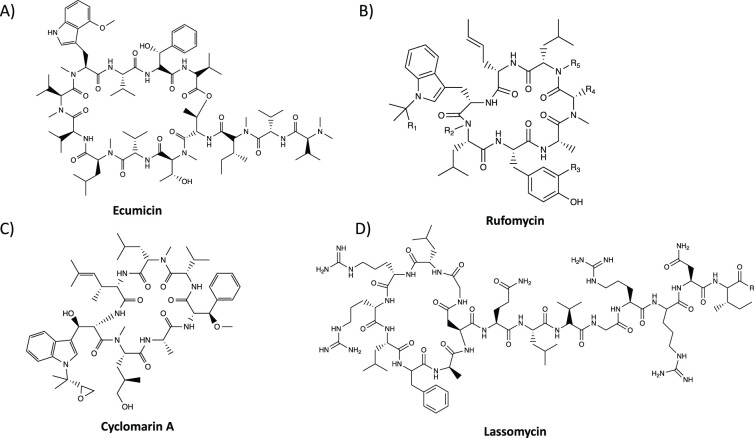
NPAs targeting the ClpC AAA+ ATPase. (**A**) Ecumicin [64]. (**B**) Rufomycin [42]. (**C**) Cyclomarin A [65,66]. (**D**) Lassomycin [67]. Chemical structures drawn using ChemDraw.

**Figure 6.**
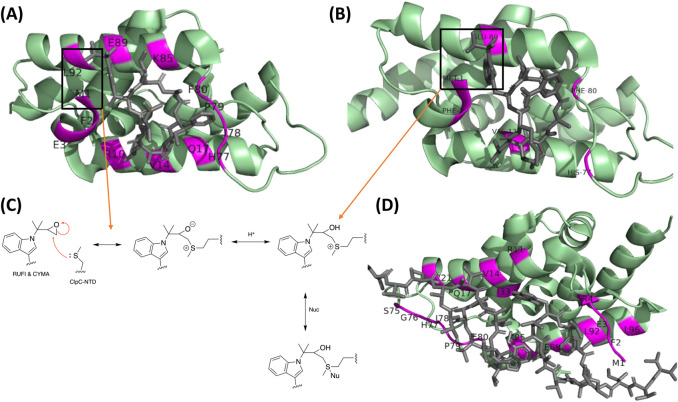
NPAs bound the to the N-terminal Domain of the Mtb ClpC1 ATPase. (**A**) Cyclomarin A bound to ClpC1 with key amino acid residues shown [66]. (**B**) Rufomycin bound to ClpC1 with key amino acid residues shown [44]. (**C**) Covalent bond formation between Met1 in ClpC1 and the epoxide substituent on Cyclomarin A and Rufomycin. (**D**) Two molecules of ecumicin bound to the ClpC1 active site with key amino acid residues shown [45]. All structures were drawn with ChemDraw and Pymol software.

The existence of NPAs has been known for many years, yet only recently has a mechanistic understanding of those targeting ATPases started to emerge. These NPAs manipulate a bacterium's own degradation system and trigger cell death through aberrant protein degradation. In this review: lassomycin, ecumicin (ECU), rufomycin I (RufI) and Cylomarin A (CymA) will be discussed, which have all been observed to bind to *M. tuberculosis* ClpC1; RufI has also been observed to bind in other mycobacteria [42] (Figure 5). X-ray crystallographic studies of the NPAs binding with ClpC1_NTD_ have been used to provide mechanistic insights into their mode of operation [43] (Figure 6).

RufI and CymA have overlapping binding positions in the hydrophobic pocket of ClpC1_NTD_. It has been shown that the formation of a covalent bond between the Met1 of ClpC1_NTD_ and epoxide group of both antibiotics is important for the inhibition of ClpC1 activity [44] (Figure 6C). On the other hand, ECU has important interactions with the carbonyl group of Met1, Leu92 and Leu96 [45]. There is still a lack of understanding as to how these interactions affect the conformational changes of ClpC1 and its interactions with ClpP. ECU, RUFI and lassomycin appear to activate ATPase activity and inhibit proteolysis by uncoupling ClpC1 from ClpP1 and ClpP2, however RufI seems to have no significant effect on ATPase activity [42]. On the other hand, CymA is believed to cause activation of the ATPase, (compared with other peptides) leading to uncontrolled proteolytic degradation while bound to ClpP. It is only recently that a molecular understanding of the conformational changes CymA imposed on ClpC has been elucidated. ClpC exists in equilibrium between its active hexamer state and its inactive decamer state and the effect CymA has on this equilibrium was probed. Initially, this was tested with the *S. aureus* proteins, where the whole N-terminal domain of *S. aureus* ClpC was replaced with that of *M. tuberculosis* [46]. *S. aureus* was used since it was believed at that time that no adapter proteins were present in *M. tuberculosis*, which was needed to compare the mechanism of action with CymA. It was found through size exclusion chromatography (SEC) and static light scattering, that when CymA was bound to ClpC_NTD_ a larger complex was formed than the inactive decamer state with lower elution volumes and mass of 2237 kDa [46]. This was surprising as CymA was expected to increase the activity of the ClpC_NTD_; instead it triggered the formation of a species corresponding in size to a 24-mer. However, SEC analysis with ClpP showed that it co-eluted with the complex and was still actively degrading proteins [46]. This is comparable to the assembly of *B. subtilis* ClpC later reported in the aforementioned BacPROTAC study. It was concluded that four ClpC_NTD_ could be present in the structure as the sum of their individual mass is equivalent of the mass of the complex observed [34]. A similar experiment, with CymA and *Mtb*ClpC1, yielded comparable observations via SEC and multiangle light scattering, confirming the formation of the supercomplex [47].

Four ClpC1 bind together in a tetrahedral arrangement through their MD regions and because their AAA2 domains are projecting outwards, they can bind with ClpP1P2 and cause uncontrolled proteolytic degradation. However, it is still unclear how CymA binding to the NTD of ClpC1 affects the MD region given the lack of close proximity; this warrants further investigation.

Finally, it has been observed that Leu92 and Leu96 in particular, as well as Val 13, His77 and Phe80, are key residues for interacting with ECU and RufI [45]. It was found that the mutations Leu92Ser and Leu96Pro resulted in 80-fold weaker binding of ECU than with wild type ClpC1. However, this was not the case with RufI, which showed a similar Kd to with the wild type [45]. Similarly, mutations that weaken RufI binding do not concomitantly weaken ECU binding. This lack of cross-resistance suggests that if resistance did emerge to a particular NPA, other NPAs can still exhibit a bactericidal effect [44].

## Acyldepsipetides target the ClpP system

ADEPs are a class of natural antibiotic compounds which were originally discovered in *Streptomyces hawaiiensis* in the 1980s [48]. They were first identified as a natural product complex of eight ADEPs dubbed A54556, the main component of which later became known as ADEP1 [49,50]. The ADEPs were found to operate through the dysregulation and aberrant activation of the ClpP protease [50]. ADEP1 was used as a framework on which to build ADEPs with improved activity and physicochemical properties. They are now made synthetically and their mechanism of action has been explored in detail over the past decade [51].

**Figure 7.**
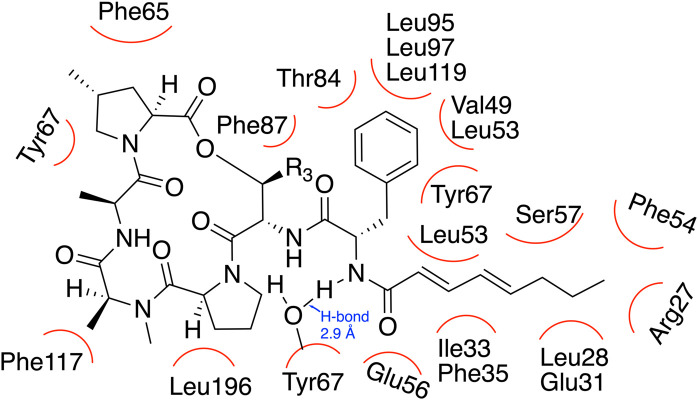
Natural ADEP binding site in ClpP with key residues interacting with the peptide drug. The red curved lines represent hydrophobic interactions between ADEP and amino acid residues in ClpP. All structures were drawn with ChemDraw software and diagram is adapted from ref. [55].

ADEP binding has been found to trigger reorientation of the ClpP sub-units and in turn affect the hydrophobic interaction between the N-terminal regions close to the axial pore of ClpP. Hydrophobic amino acids such as Pro4 and Val6 are brought into close proximity to Phe49 and Leu24, resulting in the widening of the axial pore where proteins enter [51]. Other key amino acids interacting with ADEP are shown in Figure 7. With the widening of the pore larger substrates may enter the proteolytic chamber, and since the catalytic triad is held in the same conformation these larger substrates may now also be degraded. ADEPs result in the destruction of various key proteins. One such protein is FtsZ, which is important for bacterial cell division. Thus bacterial cell growth is halted and very long filaments without a septum are formed, as observed with fluorescent spectroscopy [52]. This stops the cell division process and consequently results in cell death [52].

**Figure 8.**
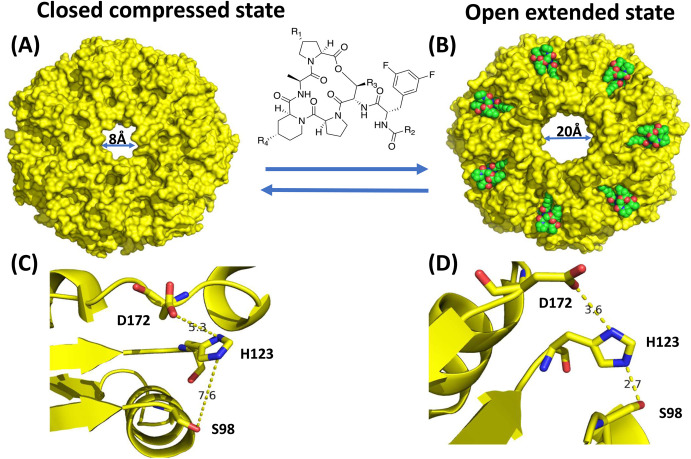
Conformational changes ADEP imposed on ClpP. (**A**) Closed state of ClpP. (**B**) ClpP open state when ADEP bound. (**C**) Catalytic triad in the closed compressed state in ClpP with hydrogen bonding distances between asp172-his123-ser98 shown. (**D**) Catalytic triad in the open extended state. All diagrams were draw with PyMol and ChemDraw software and diagram was adapted from ref. [49].

Binding of ADEPs also prevents interaction of ClpP with its cognate ATP-driven chaperone systems, as it binds directly to the same Ile-Gly-Phe/Lys binding loop, which is necessary for interaction with ClpP [51]. Recent work showed that ADEPs also allosterically control the ClpP action [53]. Mutation of Asp172Asn, one of the key residues in the catalytic triad responsible for protein degradation, brings ClpP into a compact structure, preventing protein degradation. However, upon ADEP binding proteolytic activity is regained, showing that ADEP has an allosteric effect [53]. This was confirmed by small angle X-ray scattering (SAXS) and dynamic light scattering (DLS) which suggested that the assemblies of Asp172Asn and wild type hadn't changed oligomerization state, which implied that ADEP instead caused changes in conformation [53]. When ADEP binds to the wild type and mutant, ClpP changes its conformation from a compressed conformation, where the catalytic triad residues are inactive, to an extended conformation. In the compressed conformation the hydrogen bond distances are too great for interactions to occur, whilst in the extended conformation the hydrogen bonds are closer to each other, thus enabling catalytic activity (Figure 8).

*M. tuberculosis* bacteria have a more complicated degradation system with ClpP existing in two isoforms ClpP1 and ClpP2. It appears that ADEP can only bind to ClpP2, which has a knock-on effect causing pore widening in ClpP1, showing that these drugs can also be effective against more complex protein degradation systems [54].

Even though these antibiotics have potential, their physicochemical and pharmacokinetic profiles are poor and they are only effective against Gram-positive species. This selectivity in targeting Gram-positive bacteria is not well understood, however, in a recent study scientists synthesised a large library of ADEPs varying different substituents and found that ADEPs with long non-polar linear side chains were effective against Gram-positive and Gram-negative species [55]. The binding pocket in the Gram-negative species tested contained Ser57 and Glu31, polar residues, which prevents interaction with polar aliphatic side-chains [55] (Figure 7). It is believed that having long hydrophobic acyl side chains will enhance the compatibility of these drugs with Gram-negative species, as they better complement the active site. In terms of metabolic stability, the originally extracted ADEP1 was found to be metabolically very unstable, therefore if clinical trials are to be performed metabolic stability and pharmacokinetic life have to be increased. It has been found that the acyl chain is key for the activity of this antibiotic as it is believed to anchor it in the hydrophobic site of the ClpP. Therefore, a recent study attempted to modify this chain, as while it is important for the activity of the antibiotic it also causes it to be unstable. Multiple substitutions were made and it was found that urea substitution with a phenyl group in the R_2_-position (see Figure 8 for the position of R) caused an increased half-life and decreased minimum inhibitory concentration (MIC) [56]. This is believed to be because of Tyr 63 forming three key intramolecular hydrogen bonding interactions, (as opposed to only two with ADEP 4) with the most important being with urea NH [56]. However, further optimisation of the structure still need to be performed with urea group at the R_2_ site to increase the half-life of the drug.

Finally, one of the growing problems with antibiotic resistance, is the increase in persister cells and spores in bacteria. This is a small population of bacteria, which are multidrug resistant, as these dormant cells inactivate their targets and then replicate once the stress condition is removed [57]. In deep-seated mouse thigh infections it was found that conventional antibiotics reduced the infection but did not kill it even after prolonged treatment at 24 and 48 h [57]. However, when ADEP was combined with the antibiotic drug rifampicin the infection was eradicated in 24 h and no trace of the tumour was visible [57]. On the other hand, sole treatment with rifampicin and vancomycin reduced but did not clear the infection [57]. This could show that ADEPs combined with strong conventional antibiotics such as rifampicin, could be a key solution to combat antibiotic resistance. However, even though ADEP shows very potent activity even against persister cells, further optimisation of their pharmacokinetic and metabolism profiles is necessary before they can be implemented in clinical trials.

## Targeting the sporulation mechanism

**Figure 9.**
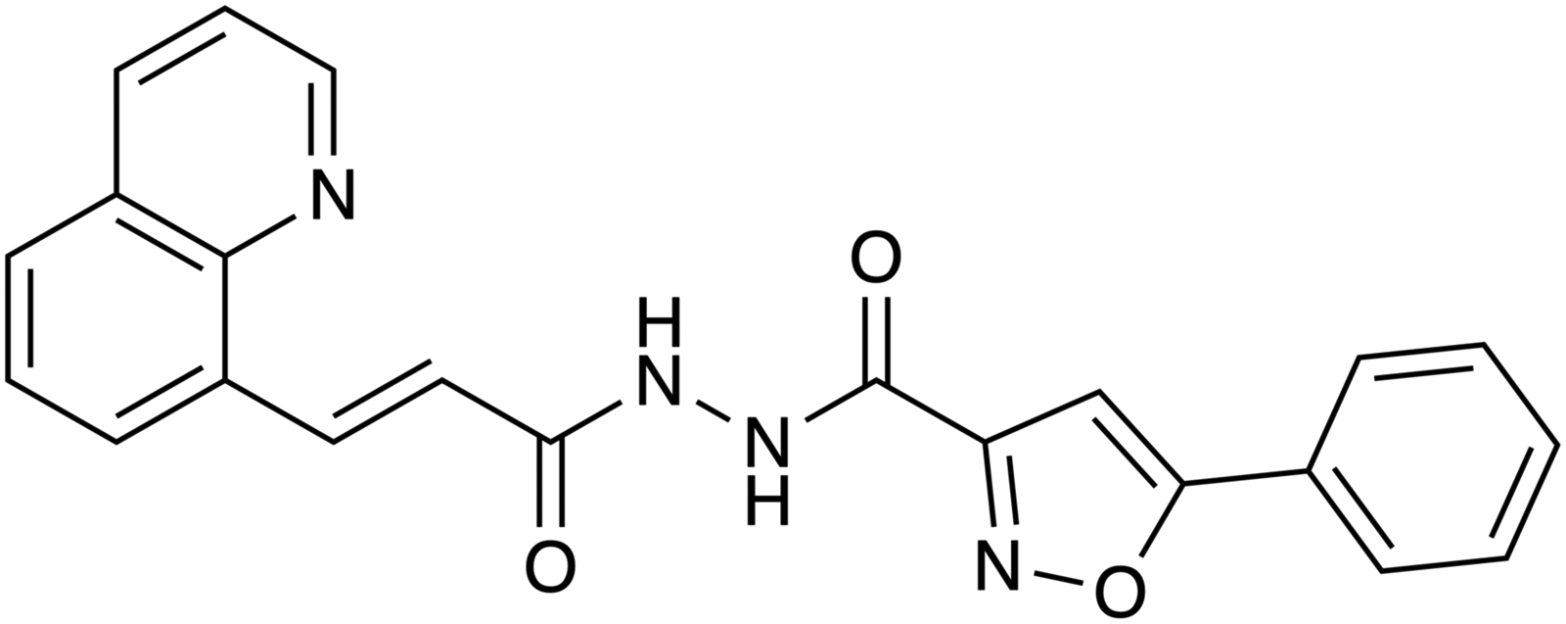
The process of sporulation in Gram-positive bacteria with the different sigma factors responsible for forespore and mother cell development during the process. All diagrams were drawn in Biorender and diagrams were reproduced from references [51,52].

Even though the targeted protein degradation system in bacteria seems a very promising target for future antibiotics, in the last five years scientists have also attempted to target a vital mechanism in bacteria called sporulation. Sporulation is a mechanism by which bacteria are able to form resilient spores in response to unfavourable conditions, allowing them to stay dormant until the environment becomes more hospitable [58]. This is a complex process and much of what is known today has been derived from studies of *B. subtilis*, (mechanistically reviewed in [58]) (Figure 9). Sporulation is tightly controlled by a series of sigma factors (σ^F^, σ^G^, σ^E^ and σ^K^) and their corresponding anti-sigma factors that regulate the expression of whole swathes of genes as the bacterium proceeds through the different stages of forespore development [59,60]. These stages include: asymmetric cell division, engulfment, lysis of the mother cell, and ultimately germination of the endospore [61]. It has been determined in *Bacillus anthracis* (Bac), the causative agent of anthrax disease, that the ClpC ATPase is essential for spore formation. Fluorescent microscopy has revealed that deletion of the chaperone results in the entrapment of the bacterial spores in their vegetative state. SpoIIAB, which is activated by the transcription factor σ^F^, and prevents forespore development, was found to be up-regulated [59]. Therefore, it may be speculated that targeting the sporulation mechanism through ClpC with NPAs, ADEPs and PROTACs could destroy the bacteria. Meanwhile, it has also been found that in *B. anthracis* that the disruption of the ClpXP proteolytic complex triggers heightened susceptibility to the effect of standard antibiotics, such as penicillin and LL-37. This was determined by mutating the IGF/L loop amino acids, which prevented ClpP interacting with ClpX [62]. This resulted in elevated hydrophobicity of the cell wall and a decrease in cell wall thickness from 59 to 55 nm, increasing access for standard antibiotic drugs [62]. A similar trend has been observed with ADEPs and rifampicin, where a combined treatment results in more efficacious bacterial eradication. Therefore, this may hypothetically represent a viable strategy in tackling sporulating pathogens until newer, more efficient therapeutics are developed in the future.

## Conclusion

Targeted protein degradation is an advancing research area with many important discoveries in the last decade moving the field in a new direction for antibiotic development. This method of killing bacteria seems promising, as the bacterium's own protein degradation system is harnessed to destroy it. This may present a more robust antimicrobial strategy against which bacteria are less able to develop countermeasures resulting in antibiotic resistance. NPAs and ADEPs are promising antibiotics, which have good permeability into bacterial cells, low MICs and high specificity for the ClpC ATPase and ClpP peptidase, respectively, making them effective candidates for modulating protein degradation. However, poor pharmacokinetic profile of ADEPs as well as lack of understanding of the mechanism of action of many NPAs are barriers that have to be overcome before the field can be extended to antibiotic drug development. Nevertheless, new pioneering technologies, such as BacPROTACs, which hijack the bacteria's own degradation mechanism for self-destruction, have now been employed in bacteria. With high efficiency and specificity, these chimeric molecules appear effective against *M. smegmatis* and *S. aureus* representative of Gram-positive and Gram-negative species. However, future developments of using these PROTACs to target endogenous proteins are essential for determining the bactericidal effect of these compounds in bacteria and further investigations are underway, including with machine learning [63].

Further research and understanding in this area is needed, yet these compounds targeting bacterial protein degradation show promise in tackling the growing threat of hospital superbugs.

## Abbreviations

AMR: antimicrobial resistance
ClpC_NTD_: N-terminal domain of ClpC
ECU: ecumicin
MD: middle domain
NPAs: natural product antibiotics
NTD: N-terminal domain
POI: protein of interest
PROTACS: proteolysis-targeting chimeras
PTB: phosphotyrosine binding domain
SEC: size exclusion chromatography
SH2: Src homology 2
